# Islets in the body are never flat: transitioning from two-dimensional (2D) monolayer culture to three-dimensional (3D) spheroid for better efficiency in the generation of functional hPSC-derived pancreatic β cells in vitro

**DOI:** 10.1186/s12964-023-01171-8

**Published:** 2023-06-22

**Authors:** Abdoulaye Diane, Layla Ibrahim Mohammed, Heba H. Al-Siddiqi

**Affiliations:** Diabetes Research Center, Qatar Biomedical Research Institute (QBRI), Hamad Bin Khalifa University (HBKU), Qatar Foundation (QF), Doha, Qatar

**Keywords:** β cells, hPSC, Differentiation, Two-dimension (2D), Three-dimension (3D)

## Abstract

Diabetes mellitus (DM), currently affecting more than 537 million people worldwide is a chronic disease characterized by impaired glucose metabolism resulting from a defect in insulin secretion, action, or both due to the loss or dysfunction of pancreatic β cells. Since cadaveric islet transplantation using Edmonton protocol has served as an effective intervention to restore normoglycaemia in T1D patients for months, stem cell-derived β cells have been explored for cell replacement therapy for diabetes. Thus, great effort has been concentrated by scientists on developing in vitro differentiation protocols to realize the therapeutic potential of hPSC-derived β cells. However, most of the 2D traditional monolayer culture could mainly generate insulin-producing β cells with immature phenotype. In the body, pancreatic islets are 3D cell arrangements with complex cell–cell and cell–ECM interactions. Therefore, it is important to consider the spatial organization of the cell in the culture environment. More recently, 3D cell culture platforms have emerged as powerful tools with huge translational potential, particularly for stem cell research. 3D protocols provide a better model to recapitulate not only the in vivo morphology, but also the cell connectivity, polarity, and gene expression mimicking more physiologically the in vivo cell niche. Therefore, the 3D culture constitutes a more relevant model that may help to fill the gap between in vitro and in vivo models. Interestingly, most of the 2D planar methodologies that successfully generated functional hPSC-derived β cells have switched to a 3D arrangement of cells from pancreatic progenitor stage either as suspension clusters or as aggregates, suggesting the effect of 3D on β cell functionality. In this review we highlight the role of dimensionality (2D vs 3D) on the differentiation efficiency for generation of hPSC-derived insulin-producing β cells in vitro. Consequently, how transitioning from 2D monolayer culture to 3D spheroid would provide a better model for an efficient generation of fully functional hPSC-derived β cells mimicking in vivo islet niche for diabetes therapy or drug screening.

Video Abstract

**Supplementary Information:**

The online version contains supplementary material available at 10.1186/s12964-023-01171-8.

## Introduction

Diabetes mellitus is a chronic and complex metabolic disorder that results from a defect in insulin secretion, action, or both [1]. According to the international diabetes federation (IDF) report in 2021, it is estimated that 537 million adults (20–79 years) worldwide are living with diabetes (www.idf.org). Approximately 10% of diabetic patients are diagnosed with type 1 diabetes (T1D), while 90% are diagnosed with type 2 diabetes (T2D). In T2D, pancreatic β cells cannot produce enough insulin to maintain glucose homoeostasis [2] and it is managed with a combination of medications and a change in lifestyle. However, as the diseases progress patients might require insulin injections [3]. T1D, also known as insulin-dependent diabetes, is a result of a selective immune-mediated pancreatic β cell destruction leading to nearly complete deficiency of insulin production. Therefore T1D patients are dependent on life-long insulin therapy [4]. Now it is well known that both T1D and T2D commonly share a dysfunction of the pancreatic β cells that negatively impacts insulin secretion. Basically, the microvascular (e.g., retinopathy, nephropathy, neuropathy) and macrovascular (e.g., coronary heart disease, myocardial infarction) complications associated with both uncontrolled T1D and T2D are immensely costly and difficult to manage, representing a major medical and financial challenge for many countries [5, 6]. The first-line pharmacological treatment plan for T1D predominantly relies on exogenous insulin injections [4]. Whilst the exogenous insulin supplementation is considered as a life-saving treatment, it is unfortunately associated with acute hypoglycemia episodes and weight gain for many patients [7]. Cadaveric islet transplantation using Edmonton protocol has demonstrated as an effective intervention to restore normoglycaemia in T1D patients for months [8]. However, this approach is limited by its high costs and serious scarcity of cadaveric donor tissues as well as the use of immunosuppressive drugs to avoid a potential risk of tissue rejection [9]. Therefore, this treatment option cannot widely be implemented in clinical practice. One of the alternatives to resolve the problem of cadaveric islet shortage is the generation of surrogate pancreatic β cells from human pluripotent stem cells (hPSC). Human pluripotent stem cells, including embryonic stem cells (ESC) and induced pluripotent stem cells (iPSC) have the ability to be directed towards any cell types in the body due to their infinite self-renewal competency. More importantly, patient specific iPSC-derived β cells also known as autologous iPSC-derived β cells would not only help to circumvent the inadequate islet supply but also overcome allogeneic immune rejection, specially in T2D condition. However, in case of T1D, the use of encapsulation device still remains crucial to prevent autoimmune attacks against the transplanted patient’s own iPSC-derived β cells. Additionally, patient specific iPSC-derived β cells can also be used to gain a better understanding of diabetes related mutations such as the inherited monogenic diabetes as well as its progression [10]. Over the last decade, great effort has been concentrated by scientists on developing technologies and in vitro protocols to efficiently and reproducibly differentiate hPSCs into mono-hormonal insulin-producing cells with key features of bona fide mature β-like cells capable of maintaining long-term functional stability following transplantation. Most of the differentiation protocols regarding hPSC-derived β-cell generation are based on conventional two-dimensional (2D) cell culture systems mimicking normal pancreatic development. The 2D protocols based on adherent culture have been an extremely valuable tool that has provided important knowledge for many years. They offer simplified and low-cost methods for modelling human diseases in vitro. However, it is well established that current 2D differentiation models towards generation of hPSC-derived pancreatic β cells have many limitations such as lack of endocrine-to-endocrine cell interactions, disturbance of islet microenvironment. Thus, they do not mimic human islet complexity, creating a need for more physiologically relevant models. Consequently, increasing efforts were dedicated to the development of the three-dimensional culture (3D) model known to more closely recapitulate in vivo islet architecture that could fulfil the existing gap between 2D cell culture and animal models. In the present review, we will first briefly describe the pancreatic islets and β cells and they role in diabetes, second illustrate the latest progresses on the induction of hPSC-derived β cells in vitro. Lastly, we will provide detailed summary of the effect of dimensionality (2D vs 3D) on the differentiation efficiency for generation of hPSC-derived insulin-producing β cells in vitro.

### Overview of human pancreatic islet anatomy and cell population

**Fig. 1:**
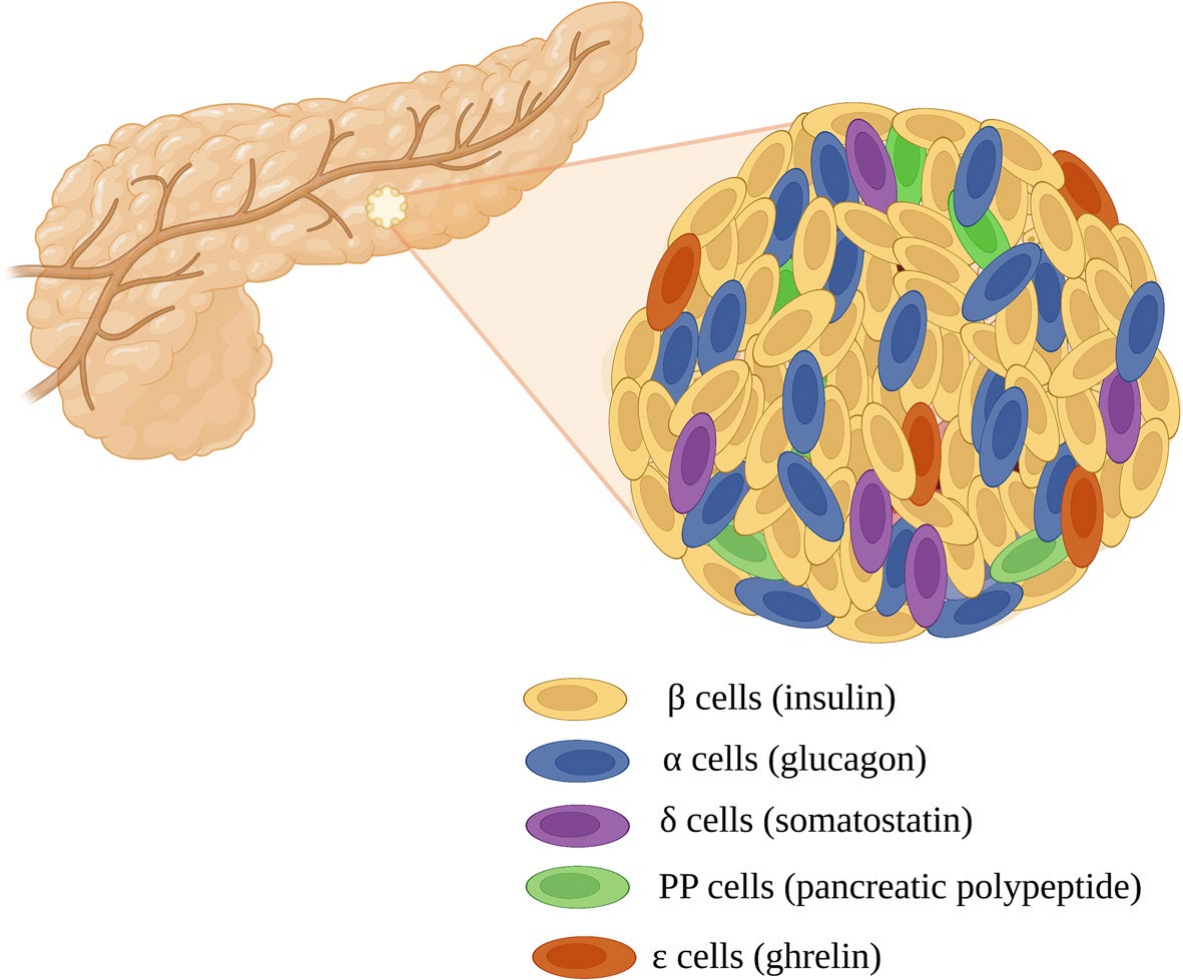
Human pancreatic endocrine islet containing different hormone-secreting cell types. Endocrine islets are composed of α, β, δ, γ cells (adapted from Hudish et al. [12])

The pancreas is a yellowish-pink gland located in the gastrointestinal tract that plays a fundamental role in the body due to its mixed exocrine–endocrine function [11]. The endocrine cells, representing less than 5% of the total pancreas mass, are constituted by mixed populations of hormone-producing cells (the islets of Langerhans). In adult human islets five major types of cells co-exist: insulin-secreting β cells, glucagon-secreting α-cells, somatostatin-secreting δ-cells, pancreatic polypeptide (PP)-secreting y-cells and recently described ghrelin-expressing ε-cells [12, 13] (Fig. 1). In the human islets, β cells represent ~ 60% of the cells and are highly intermingled with the other endocrine cells, particularly with α-cells, the second most abundant cells (~ 30%). This configuration is important for the optimal glucose homeostasis ensured by β and α cells [14]. The remaining 10%, randomly disseminated throughout the islets [15] involves δ-cells known to counteract the synthesis and secretion of both insulin and glucagon [16], PP cells and ε-cells. The anatomical organization of mammalian islet cells is remarkably heterogeneous, with variable islet size and cell type composition between species, but also showing variation from birth to adulthood and across individuals [17]. Example, the pancreas of a 8-week-old mouse possesses about 1000 islets with each islet containing ~ 800 β cells while in human pancreas there are about 100,000 islets, containing each an average 400–600 β cells [7]. The principal role of β cells is to synthesize and secrete insulin, a 51-amino acid peptide that is essential for cellular nutrient uptake. Insulin is responsible for maintaining glucose homeostasis. Glucose is an important metabolic substrate and a major source of energy for almost all mammalian cells including pancreatic β cells. One of the important characteristics of human islets is their fine-tuned synthesis and secretion of insulin in response to glucose. Pancreatic β cells sense changes in plasma glucose and adjust insulin release according to the body needs. The exocytosis of insulin is strictly controlled by glucose metabolism through glycolysis coupled with mitochondrial oxidative ATP production [18]. When glucose enters β cells, it is phosphorylated by glucokinase and converted to pyruvate by glycolysis. Pyruvate preferentially enters the mitochondria and oxidized in the mitochondrial tricarboxylic acid (TCA) cycle, generating energy in the form of ATP, and thus increasing the ATP to ADP ratio and closure of K_ATP_ channels. This results in the depolarization of the cell membrane that opens voltage-gated Ca^2+^ channels, raising the cytosolic Ca^2+^ concentration, which triggers insulin exocytosis.

### Mapping hPSC differentiation onto human pancreatogenesis

**Fig. 2:**
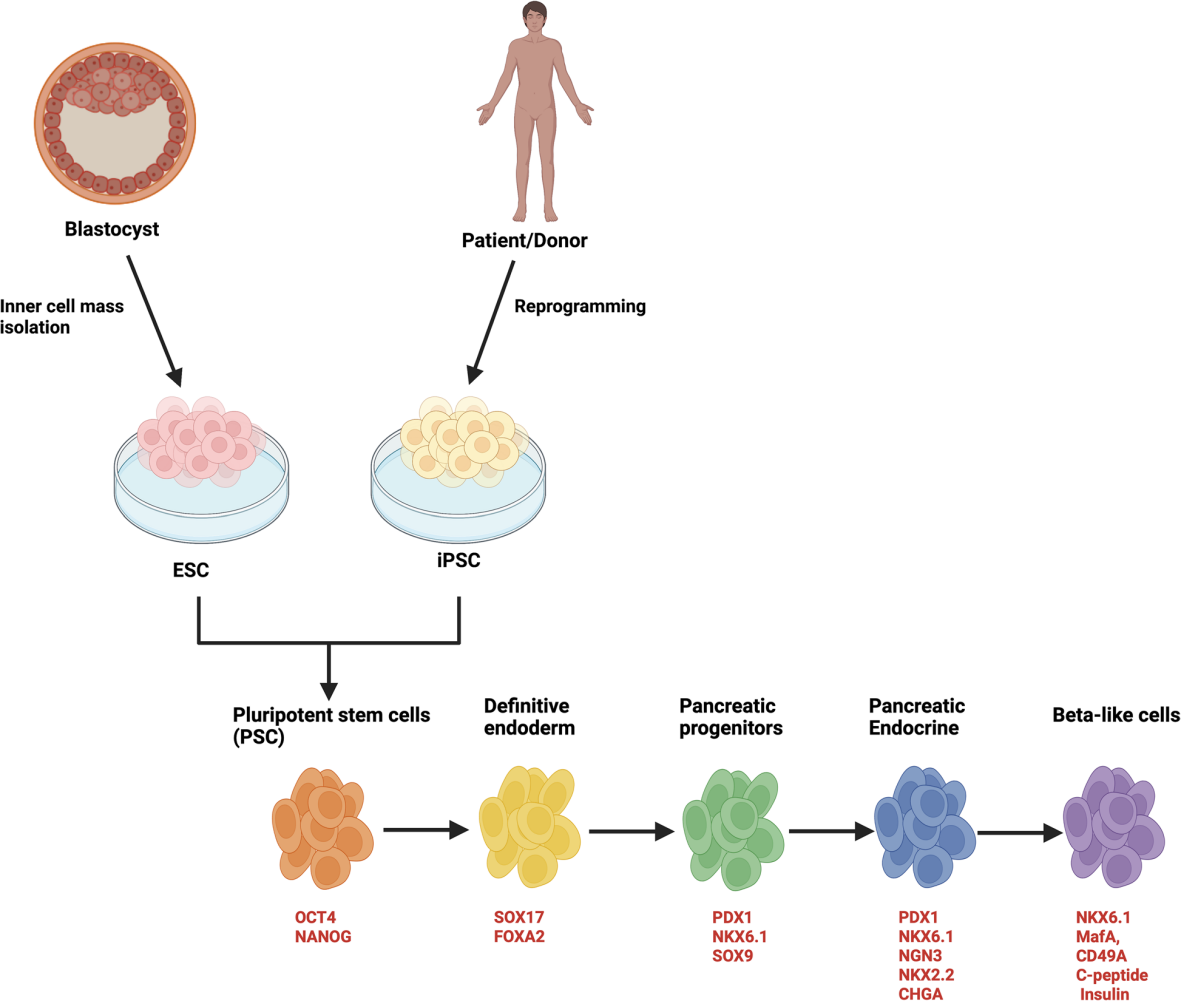
Schematic of the six-stage hPSC-derived β cell differentiation protocol [20]. Relevant-stage gene expression markers are also indicated

Pancreas organogenesis depends on interactions of endoderm-derived epithelial cells with their surrounding mesenchymal layer and also with its nearest neighbor, the chorda dorsalis and the dorsal aorta. It involves a complex coordinated cooperation of signaling events and transcriptional networks that steer a stepwise process from early pancreas specification to the final mature organ state with all different cell types proportionally balanced. At the morphological and genetic levels, there is a scarcity of information related to the development of the human pancreas. Interestingly, most of our current understanding of both human pancreas organogenesis and β cell physiology are predominantly the extrapolation from data obtained in other species, particularly rodents [19]. Information gained from those studies was used by different research groups to develop protocols for in vitro stepwise directed differentiation of stem cells towards insulin-producing β cells. Sequentially, hPSC are well-timed exposed to different cocktails of key known growth factors and small molecules, able to influence signaling pathways and transcription factors to mimic bona fide β cell development throughout different stages of pancreatogenesis. This directed differentiation protocol occurs by first inducing definitive endoderm (DE), followed by generation of primitive gut tube (PGT), posterior foregut (PF), pancreatic progenitor (PP), endocrine precursors (EP) and finally β cells (Fig. 2). Each of these different stages of the differentiation can accurately be identified with specific relevant transcription factors and other key markers using flow cytometry, immunofluorescence staining, Western blot or RT-PCR (Fig. 2).

Since the first experiments pursuing pancreatic differentiation using human ESC (H9 cells) ∼20 years ago [21], extensive in vitro differentiation protocols to generate insulin-producing β cells have been developed during the last decade and half around the pioneering publications of D’Amour et al. [22, 23]. Overall, in vitro differentiation protocols developed for generation of β cells were developed based on knowledge obtained from pancreatic organogenesis, as a recapitulation of pancreas development in vivo. Interestingly, many of these protocols used traditional two-dimensional monolayer tissue cultures, which results in robust generation of PDX1 + /NKX6–1 + pancreatic progenitors that could mature in vivo after transplantation [24]. However, 2D culture generally generated low efficiency and dysfunctional immature hPSC-derived insulin positive β cells from these progenitors. This relative immaturity of hPSC-derived β cells may reflect the poor in vivo representation of the 2D method, consistent with a greater maturity achieved following in vivo transplantation in rodents [24]. Besides the 2D environment, the gap of knowledge about the developmental niche that contains the necessary signaling factors for pancreatic differentiation may also have an impact on the immaturity of the β cells. In contrast, almost all protocols that successfully generated dynamic GSIS-responsive hPSC-derived β cells in vitro employed a three-dimensional arrangement of cells either as suspension clusters [25, 26] or as aggregates [27] from pancreatic progenitors. Therefore, the terminally differentiated β cells in the dish using 2D protocol fail to recapitulate the complexity of the pancreas niche in vivo [28, 29] when compared to the three-dimensional culture (3D). This suggests the role of dimensionality (2D vs 3D) on the differentiation efficiency as well as on the maturity and functionality of hPSC-derived insulin-producing β cells in vitro.

### Traditional monolayer 2D culture

Conventional 2D culture consisting of culturing cells directly on a rigid substrate (e.g. polystyrene or glass), usually coated with substrates that mimic extra-cellular matrix (ECM) composition and promote cell adhesion has been an extremely valuable tool for more than 100 years to model tissues or diseases [30]. The advantages of monolayer or 2D culture include simplicity, availability, and relatively low cost. Despite the unquestionable importance of the 2D culture, scientists, however, argue that it has several limitations such as abnormal cell–cell and cell-extra cellular matrix (ECM) interactions, and lack of tissue organization and architecture. Another concern with monolayer cultures is the lack of nutrients and oxygen diffusion and waste removal dynamics [31]. Therefore, these limitations were reported to negatively impact cell morphology, survival, proliferation, differentiation and functionality [32].

### 3D model for mimicking in vivo cell niche environment

**Fig. 3:**
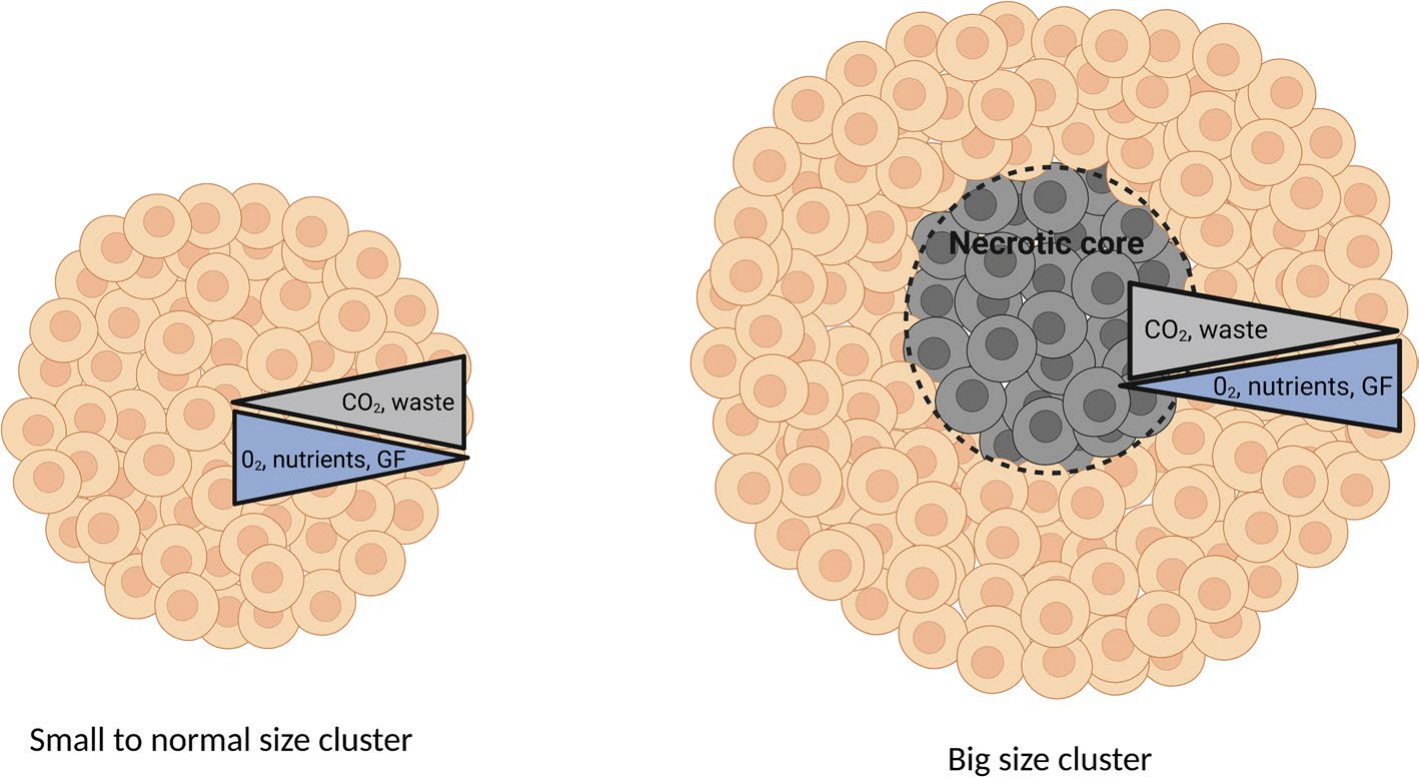
Heterogeneity in clusters generated in 3D culture showing the gradient distribution of nutrients, O_2_, CO_2_, and growth factors leading to necrotic cores as the size of the clusters increases

Dimensionality has increasingly begun to emerge as one of the critical parameters to influence a range of downstream signaling pathways within cells, such as those involving the cytoarchitecture and cell fate regulation [33]. In recent years there was a big interest in transitioning from the traditional 2D cell culture systems to more physiologically relevant 3D models for research and drug development. It is thought that cells cultured in 3D more closely mimic the in vivo cell niche and there are two main approaches to develop 3D cultures in vitro: scaffold-based and scaffold-free methods. The scaffold-based system is designed to have a fully interconnected geometry, structural integrity and a defined 3D shape allowing cell assembling into 3D clusters, which resembles the self-organization that occurs in suspension cultures. The scaffold-based system formed from synthetic biomaterials such as polyethylene glycol or polylactide-co-glycolide can serve as a supportive matrix to facilitate cell–cell interactions and promote the differentiation of stem cells toward glucose-responsive insulin-producing β cells in vitro [34]. The scaffold-free technique, contrary to scaffold-based culture, does not contain any added biomaterials to the culture medium or dish and spherical clusters (also referred to as cell aggregates or spheroids) are self-assembled using ECM produced by the cells themselves [35], therefore demonstrating development of the cellular niche that naturally occurs in vivo [36]. In contrast to 2D, 3D models promote more complex interactions between cells and provide better spatial organization and therefore represent more relevant models mimicking the in vivo organ environment. Due to these advantages, 3D protocols are considered to exhibit greater potential for the generation of hPSC-derived pancreatic β cells. Nonetheless, as the size of the clusters increases, there is a gradient distribution of components (nutrients, O_2_, CO_2_, growth factors) leading to necrotic cores, cell death or heterogeneity (Fig. 3). A summary for comparison with traditional 2D monolayer culture methods, advantages and disadvantages is shown in Table 1.

**Table 1:** Comparison between 2 and 3D protocols: Advantages and disadvantages

2D		3D
Advantages	-Simplicity of use -less expensive (cheap) -Homogenous culture -More reproducible -Easy access to cells for downstream applications such as flow, immunostaining, microscope visualization	-Promote more cell–cell interactions -Promote more cell-EMC interactions - Provide better spatial organization - More relevant model mimicking the in vivo cell/tissue/organ environment
Disadvantages	-limited cell–cell interaction -limited cell-ECM interaction -altered in vitro cell signaling networks -possible altered gene expression -lack of prediction when used in drug discovery and clinical trials -Do not recapitulate the in vivo niche	-lack of homogeneous nutrient and oxygen distribution and diffusion kinetics, leading to necrotic area, cell death and heterogeneity -expensive equipment (e.g. bioreactors) -more challenge for access to cells for downstream applications (flow cytometry, immunostaining) -more challenge for visualization, microscopy/electrophysiology techniques

Regarding the generation of hPSC-derived β cells in vitro, several methods of 3D cluster culture have been successfully developed, including magnetic spinner flasks [25, 37], 6-well plates on an orbital shaker [20, 38], manually pipetting onto trans-well culture inserts [27], honeycomb technology [39], and dialysis suspension culture system [40].

Focusing on the hPSC-derived pancreatic β cell generation in vitro, detailed comparison of all relevant stages of the differentiation process between 2 and 3D culture protocols has not been well described.

### 2D vs 3D in definitive endoderm induction in hPSC-derived β cell differentiation process

Definitive endoderm (DE) is the innermost of the three principal germ layers and is generated from ingress of endoderm precursors into the anterior primitive streak and middle streak regoins. This induces the epithelial lining of the digestive and respiratory tracts, from which arise a diverse number of different types of organs such as liver, lungs, thyroid, thymus, and pancreas [41, 42]. Therefore, DE differentiation is generally performed to prepare metabolic organ derived cells such as hepatocytes, pancreatic β cells and intestine epithelial cells [43]. Since pancreatic β cell differentiation originates with induction of definitive endoderm, DE differentiation from hPSC is the first critical process and step in differentiating hPSC-derived β cells. to uncover possible role of the dimensionality, Yabe et al. have compared the gene expression patterns between suspension (3D bioreactor spinner flask and Gyratory 6-well plates on an orbital shaker) and adherent (2D) cultured hiPSC during DE differentiation [44]. Interestingly, they demonstrated that the expression of DE marker genes (Sox17, FOXA2, HNF4a, HNF1b) are faster and more strongly induced in 3 D suspension culture than in conventional monolayer 2D culture, suggesting that 3D protocol favors DE differentiation [44]. However, using a 3D dialysis suspension culture system known to potentially refine the culture medium with continuous glucose supply, lactate removal, and autocrine factors retaining, no statistical differences were found on the expression of genes related to DE lineage markers (Sox17, FOXA2, HNF4a) between 2 and 3D methods [43]. Similar findings were reported by Wang et al. [45]. Importantly, these authors claimed that the already very high efficiency of DE induction observed in the 2D method might explain the absence of further increase in the efficiency of DE induction with the 3D protocol; suggesting that 3D method is not required for DE induction in the context of a very efficient 2D planar protocol.

### 2D vs 3D in pancreatic progenitors’ induction in hPSC-derived β cell differentiation

The key events in the stem cell differentiation toward β cells are the conversion of pancreatic progenitors (identified by expression of the pancreatic and duodenal homeobox 1 (PDX1) protein) to endocrine precursors indispensable for inducing a β cell fate. Differentiation of DE to pancreatic progenitors is controlled by PDX1 transcription factor that promotes pancreatic differentiation cooperatively with other transcription factor, such as NK6 homeobox transcription factor-related locus 1 (NKX6.1), SRY-Box Transcription Factor 9 (Sox9). Several groups have attempted to differentiate hPSC into PDX1 positive and/or PDX1 and NKX6.1 co-expressing pancreatic progenitors using 2D monolayer cultures [46, 47]. It has been demonstrated that PDX1/NKX6.1 co-expression is required for the generation of mono-hormonal, and glucose-responsive insulin-producing β cells [48]. Specifically, NKX6.1, is a β cell marker essential for its maturation and functionality [48]. Prior to the very most recent improved 2D planar method [20], reports have shown that a 3D arrangement of cells either as suspension clusters or as aggregates during the differentiation process improves the efficiency of pancreatic progenitors’ induction as compared to monolayer cultures [49]. Recently, introducing new protocols based on 3D suspension culture throughout the entire differentiation process have been reported [44, 50]. Importantly, Melton’s group has reported more than 90% PDX1^+^ pancreatic progenitors using large scale suspension 3D protocol [25, 51]. Furthermore, culturing cells as 3D aggregates increased the expression of pancreatic progenitor marker gene (PDX1) by threefold as compared to 2D [45]. Consistently, Dettmer et al. [52] found a highest expression of Sox9 (a known transcription factor for pancreatic progenitors) in 3D compared to 2D using a reporter cell line, indicating that 3D protocol enhances the efficiency of pancreatic progenitors’ induction.

### Comparison of endocrine precursor cells derived from 2D vs 3D cultures

Several transcription factors were shown to tightly regulate endocrine progenitor, including Neurogenin-3, a transcription factor capable of driving pancreatic precursors towards the endocrine cell fate [53, 54]. Using a loss-/gain-of-function approach and lineage tracing, the pathways controlling the differential selection of the endocrine fates are linked to the function of homeodomain-containing factors such as Nkx2.2, Nkx6.1, Pax4 and Arx, all of which are co-expressed with Ngn3 and act in a concerted fashion to induce endocrine progenitor cells to produce all the other endocrine hormone cell types (α, γ,δ, ε) of the pancreas [55]. In hPSC-derived β cell differentiation process, endocrine precursor cell-specific transcription factors from pancreatic progenitors can be generated in both 2D and 3D cell cultures [27, 56] by the inhibition of the Notch signaling. Notch signaling pathway is implicated in pancreatic cell-type specification. It seems critical for the decision between the endocrine and exocrine fates in the developing pancreas; so that a lack of this signaling promotes the endocrine fate (while cells with active Notch signalling adopt the exocrine fate). Blocking Notch receptor activation in early pancreatic progenitors results in early endocrine cell differentiation at the expense of pancreatic cell proliferation [57]. Moreover, mice lacking the Notch ligand Dll1 showed accelerated differentiation of endocrine cells in the pancreas [58], confirming the role of Notch signaling for proper development of pancreatic endocrine cells. To comprehensively compare 2D and 3D cultures in the efficiency of endocrine precursor generation, Xiaofang and al [59]. examined gene expression of pancreas-specific markers, and functional characteristics in 2D culture-induced endocrine precursors and 3D culture-induced endocrine precursors. They found that the mRNA expression levels of PDX1, NKX6.1, NGN3, and insulin were significantly lower in 2D culture when compared with 3D protocol. In an elegant study, Rezania et al. [27] reported that the use of air–liquid interface allowing for more basal and apical polarity [60] of cells and more exposure to atmospheric oxygen [61] to generate clusters in a seven-stage-specific differentiation approach resulted in upregulation of NGN3 mRNA (a pancreatic endocrine precursor-specific transcription factor) along with insulin, compared with planar 2D culture; suggesting that 3D method is more efficient than 2D monolayer culture in hPSC-derived endocrine precursors generation in vitro.

### Comparison of β cells derived from 2D vs 3D cultures

The first directed differentiation protocol of generating insulin-expressing β cells from hPSC in vitro was reported by D’Amour and colleagues [22, 62]. While this pioneer protocol yielded relatively a small fraction of insulin positive cells (~ 7%) using adherent 2D method, it has tremendously served as an excellent landmark for many researchers to develop and refine protocols leading to more efficient ways to generate hPSC-derived β cells over the last 15 years. However, in many of those studies using 2D protocols, a large number of polyhormonal insulin-expressing cells co-expressing glucagon and somatostatin have been observed. Detailed characterization of the β-like cells generated by this 2D culture platform showed impaired GSIS and inappropriate expression of key transcription factors of bona fide adult β cell counterparts, indicating a generation of fetal β cells that mature in vivo after transplantation. Pancreatic islets are 3D arrangements of cells with intricate cell–cell and cell–ECM interactions. Thus, it is important to consider the spatial organization of the cell in the culture environment. More recently, 3D cell culture platforms have emerged and demonstrated advantages as they recapitulate mechanical and biochemical stimuli present in native tissue [63]. Since then, subsequent differentiation protocol modifications aimed at improving stem cell-derived β cell function have utilized a three-dimensional arrangement of cells either as suspension clusters [25, 26] as aggregates [27] to generate functional and terminally differentiated β cells. Importantly, it has been demonstrated that clustering/reaggregation of immature β-like cells is a critical step in maturation and generation of fully functional hPSC-derived β cells in vitro [64]. Therefore, most of the 2D protocols that successfully generated glucose-responsive hPSC-derived β cells in vitro that secreted high amounts of insulin along with expression of mature β cell markers utilized a 3D arrangement from the pancreatic progenitor’s stage [27]. Additionally, Xiaofang et al. [59] reported that 3D culture increased the differentiation efficiency (23.7% vs. 16%) and led to the generation of monohormonal endocrine cells, while in 2D, insulin positive cells also co-express glucagon and somatostatin (termed as “polyhormonal” cells). These 3D-derived β cells responded more sensitively to glucose, potassium chloride and Forskolin than 2D-derived β cells. At the transcriptomic level, 3D culture leads to a higher expression of β cell markers such as PDX1, NKX6.1 and MAFA compared with 2D [59, 65]. Recently, Dettmer and al. knocked-in mCherry into the human INS-locus and next compared the number of mCherry^+^/INS^+^ cells obtained in 2D vs. 3D. Interestingly, 3D orbital shaking culture yielded significantly more mCherry^+^/INS^+^ cells associated with higher insulin and c-peptide content and increased expression of glucose-sensing apparatus marker genes GCK, KIR6.2, SUR1 and Glut2 compared to 2D condition [52]. They also found improved insulin-releasing properties in stem cell-derived β cells from 3D as compared to 2D [52]. All these findings suggest that 3D culture promotes the functional maturation of hPSC-derived β cells mimicking the specificity of native islet with greater physiological relevance than conventional 2D culture.

## Conclusion

Due to the opportunity offered by stem cell-derived β cells for cell replacement therapy for diabetes, substantial progress has been made in the differentiation of hPSCs into pancreatic β cells over the last two decades. Since pancreatic islets are 3D cell arrangements with complex cell–cell and cell–ECM interactions, protocols using 3D showed quantitatively (high % of β cells) and qualitatively (less % of polyhormonal cells) a higher efficiency in generation of hPSC-derived β cells when compared to conventional 2D culture. Therefore, transitioning from 2D monolayer culture to 3D spheroid method that mimics in vivo islet niche would lead to substantial phenotypic improvement of hPSC-derived β cells for diabetes therapy or drug screening.

## Data Availability

Not applicable.

## Abbreviations

T1D: Type 1 diabetes
T2D: Type 2 diabetes
2D: 2 Dimension
3D: 3 Dimension
hPSC: Human pluripotent stem cells
Oct4: Octamer-binding transcription factor 4
Nanog: Nanog Homeobox
Sox17: SRY-Box Transcription Factor 17
Sox9: SRY-Box Transcription Factor 9
FOXA2: Forkhead box protein A2
HNF4a: Hepatocyte nuclear factor 4-alpha
HNF1b: Hepatocyte nuclear factor 1-beta
PDX1: Pancreatic And Duodenal Homeobox 1
NKX6.1: NK6 homeobox transcription factor-related locus 1
Nkx2.2: NK6 homeobox transcription factor-related locus 2
NGN3: Neurogenin-3
ISL1: Insulin gene enhancer protein ISL-1
MAFA: MAF BZIP Transcription Factor A
Pax4: Paired box 4
CHGA: Chromogranin A
Arx: Aristaless related homeobox
ECM: Extra cellular matrix
GSIS: Glucose-stimulated insulin secretion
